# Comparison Therapeutic Effects of Ciprofloxacin, Silver Nanoparticles and Their Combination in the Treatment of *Pseudomonas* keratitis in Rabbit: An Experimental Study

**Published:** 2019

**Authors:** Mohammad Zavarshani, Malahat Ahmadi, Habib Dastmalchi Saei, Ali Asghar Tehrani, Bahram Dalir Naghadeh

**Affiliations:** a *Microbiology Department, Faculty of Veterinary Medicine, Urmia University, Urmia, Iran.*; b *Pathobiology Department, Faculty of Veterinary Medicine, Urmia University, Urmia, Iran. *; c *Clinical Sciences* *Department, Faculty of Veterinary Medicine, Urmia University, Urmia, Iran.*

**Keywords:** Ciprofloxacin, Keratitis, Pseudomonas, Rabbit, Silver

## Abstract

*Pseudomonas aeruginosa* is one of the most common causes of keratitis. The current study was done to evaluate the therapeutic effects of antibacterial combinations with Silver nanoparticles (Ag-NPs) and Ciprofloxacin in experimental *Pseudomonas* keratitis. Sixty four New Zealand rabbits were prepared. All rabbits were randomly categorized into eight groups (each group containing eight rabbits): Control +, Control −, Ciprofloxacin, Ag-NPs, Ciprofloxacin plus Betamethasone, Ag-NPs plus Betamethasone, Ciprofloxacin plus Ag-NPs, and Ciprofloxacin plus Ag-NPs plus Betamethasone. Twelve hours after bacterial inoculation into the cornea, the eyes were examined daily to evaluate the number of days of ocular discharge and blepharospasm. Also, after 108 and 204 h, first grading of corneal opacity was done and then four rabbits of each groups were euthanized for bacterial count. The results showed that the means of days of blepharospasm, ocular discharge, and bacterial counts (log CFU mL^-1^) were significantly different in the treatment groups at 108 and 204 h (*P *<0.0005, ANOVA). According to Tukey’s test, Ciprofloxacin plus Ag-NPs plus Betamethasone group was significantly less than Control +, Ag-NPs, and Ag-NPs plus Betamethasone groups for these variables (*P* < 0.05). The mean rank of opacity scores was significantly different between treatment groups (*P *= 0.01, Kruskal-Wallis). Mann-Whitney U-test revealed that Ciprofloxacin plus Ag-NPs plus Betamethasone group had significantly better score than Control +, Ag-NPs, and Ag-NPs plus Betamethasone groups (*P *< 0.05). It seems Ag-NPs can be an appropriate adjuvant for Ciprofloxacin, but due to the results they can’t be an alternative for Ciprofloxacin to treat *Pseudomonas* keratitis.

## Introduction

Bacterial keratitis is a vision threating ocular infection. A large number of bacteria are present in the eye which includes *Staphylococcus sp*, *Corynebacterium sp*, and *Streptococcus* *sp* (1). However, many other outside bacteria such as *Pseudomonas aeruginosa* can make tragic diseases and unfavorable results (2). *Pseudomonas* *aeruginosa* is a Gram-negative and ubiquitous bacteria that causes severe infections especially opportunistic infection in patients with immune deficiency (3). Recently, eye infections caused by *Pseudomonas* *aeruginosa* especially in hospitalized people with systemic diseases have been impressive (4). *Pseudomonas* keratitis occurs often followed by severe corneal ulceration or perforation (5). This infection can progress rapidly and lead to vision loss or complete blindness (6). Third and fourth generation of Fluoroquinolones such as Ciprofloxacin and Ofloxacin are used as choice drugs for the treatment of bacterial keratitis, especially *Pseudomonas* keratitis (7). *Pseudomonas* *aeruginosa* can acquire antibiotics resistance in several ways (3). So, the emergence of *Pseudomonas* resistant strains to antibiotics isn’t unexpected particularly using just one type of antibiotic (8). According to the increasing antibiotic resistant infections in hospitals, the development and application of the alternatives to antibiotic are necessary (9). From centuries metals such as silver, copper, gold, and aluminum have been used for treating wound infections (10, 11). More than 2000 years ago, the therapeutic properties of Silver have been known (12). Ag-NPs are toxic to bacteria at low concentrations. Also, these particles have anti-viral, anti-fungal, and anti-inflammatory effects (13). Several *in-vitro* studies have been done on the combination of antibiotics and metal nanoparticles against Gram-positive and Gram-negative bacteria. The results showed that they have synergistic effect on bacteria but Gram-negative bacteria were more susceptible than Gram-positive (14). This biological feature can be useful in the rapid treatment of bacterial infections such as *Pseudomonas* keratitis. In the present study, we compared the effects of eight different treatment combinations in the treatment process of *Pseudomonas* keratitis. 

## Experimental

*Animals*

Sixty eight New Zealand male rabbits with weighting 1.5-3 kg were obtained from Razi institute, Tehran, Iran. The animals were fed with water and alfalfa hay. All animals were examined in terms of eye infection before starting the study. The infected animals were treated according to the guidelines of the association for research in vision and ophthalmology.

*Silver nanoparticles, Ciprofloxacin and Betamethasone preparation*

Solution of Ag-NPs with 40-50 nm size and 4000 mg l^-1^ concentration was prepared (Malvern, USA). Also, Ciprofloxacin 3%, and Betamethasone 1% in the form of eye drops were purchased from drugstore (Sina Daru, Iran). Serial dilutions of Silver nanoparticles, Ciprofloxacin, and their combination were done by Muller Hinton broth (Merck, Germany) with the same mixture in the ratio 1:1 to determine the MIC and MBC. Then, 0.2 mL of bacterial suspension (10^5^ CFU mL^-1^) was inoculated into the tubes containing different concentration of them (15). 

**Table 1:** MIC and MBC of Ciprofloxacin (µg mL-1) Ag-NPs (mg L-1) and their combination

**Group**	**MIC**	**MBC**
Ciprofloxacin	5.85	11.71
Silver nanoparticles	15.62	31.25
Ciprofloxacin + Silver nanoparticles	0.73	1.46
	0.97	1.95

**Table 2:** The details of Kruskal-Wallis test of corneal opacity scores (Mean rank) among all treatment groups

	**108 h**	**204 h**
Chi-Square	25.422	26.647
df	7	7
Asymp. Sig.*	0.001	0.000

* *P *value < 0.05

**Table 3:** Comparison of corneal opacity scores (Mean rank) between two groups

**Group**	**108 h**	**204 h**
**CO +**	28.00c	28.00b
N	26.00c	26.00b
N+B	22.00c	24.00b
C+N+B	10.50b	7.50c
C+B	13.38bc	13.63c
C	16.25bc	15.75c
C+N	13.38bc	13.63c
CO −	2.50a	3.50a

Insignificant differences (*P *> 0.05) between different treatments are shown by different letters (a, b, c and d) in each group.

**Table 4:** The details of ANOVA test of number of days of blepharospasm and ocular discharge (Mean ± SD) among all treatment groups

**Variable Time**	**Blepharospasm 108 h**	**Ocular discharge 108 h**	**Blepharospasm 204 h**	**Ocular discharge 204 h**
Sum of Squares	54.969	54.875	191.5000	168.000
df	7	7	7	7
F	251.286	188.43	82.071	57.600
Sig.*	0.000	0.000	0.000	0.000

* P value < 0.05

**Table 5:** Comparison of number of days of blepharospasm and ocular discharge (Mean ± SD) between two groups

**Group**	**Blepharospasm 108 h**	**Ocular discharge 108 h**	**Blepharospasm 204 h**	**Ocular discharge 204 h**
CO +	4.00 ± 0.00c	4.00 ± 0.00c	8.00 ± 0.00f	8.00 ± 0.00e
N	4.00 ± 0.00c	4.00 ± 0.00c	7.25 ± 0.50ef	7.25 ± 0.50de
N+B	4.00 ± 0.00c	4.00 ± 0.00c	7.00 ± 0.00def	7.00 ± 0.00cde
C+N+B	3.25 ± 0.50b	3.50 ± 0.57b	3.25 ± 0.50b	4.00 ± 0.81b
C+B	4.00 ± 0.00c	4.00 ± 0.00c	5.75 ± 1.25cd	5.50 ± 1.29bcd
C	4.00 ± 0.00c	4.00 ± 0.00c	6.50 ± 0.57cde	6.25 ± 0.50cd
C+N	4.00 ± 0.00c	4.00 ± 0.00c	5.25 ± 0.50c	5.75 ± 0.50cd
CO −	0.00 ± 0.00a	0.00 ± 0.00a	0.00 ± 0.00a	0.25 ± 0.50a

Insignificant differences (*P *> 0.05) between different treatments are shown by different letters (a, b, c, d, e and f) in each group.

**Table 6:** The details of ANOVA test of bacterial counts (log CFU mL-1) (Mean ± SD) among all treatment groups

	**108 h**	**204 h**
Sum of Squares	341.469	634.969
df	7	7
F	67.870	129.972
Sig.*	0.000	0.000

* *P *value < 0.05

**Table 7:** Comparison of bacterial counts (log CFU mL-1).(Mean ± SD) between two groups

**Group**	**108 h**	**204 h**
CO +	11.25 ± 0.92d	15.00 ± 0.81d
N	6.50 ± 1.29c	6.50 ± 1.29c
N+B	7.50 ± 1.29c	6.50 ± 1.29c
C+N+B	2.50 ± 0.57ab	1.50 ± 0.57ab
C+B	3.50 ± 0.57b	2.50 ± 0.57b
C	3.50 ± 0.57b	2.50 ± 0.57b
C+N	2.75 ± 0.50b	1.75 ± 0.50ab
CO −	0.75 ± 0.50a	0.50 ± 0.57a

Insignificant differences (*P *> 0.05) between different treatments are shown by different letters (a, b, c and d) in each group.

**Figure 1:**
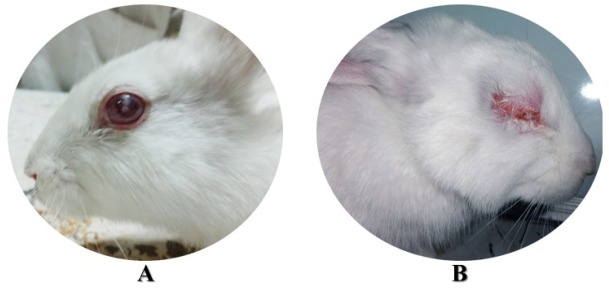
Dense opacity (A) blepharospasm and ocular discharge (B) 12 and 48 h after bacterial inoculation, respectively

**Figure 2:**
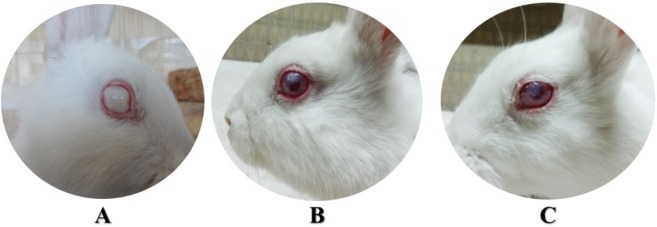
Different scoring of corneal opacity in groups CO + (A), C+N+B at 108 h (B) and 204 h (C)

*Induction of experimental keratitis*

*Pseudomonas* *aeruginosa PAO1* was obtained from McMaster University, Canada by Dr. Nima Hoseini Jazani, School of Medicine, Urmia University. 

The Muller Hinton agar (Merck, Germany) medium was used for bacterial growth. *Pseudomonas* keratitis was created by Suspension 0.5 McFarland of bacteria. After standardization, the concentration of bacterial suspension changed to 10^5^ CFU mL^-1^ (5). For experimental induction keratitis the rabbit^’^s immune system was weakened by Azathioprine (Ramopharmin, Iran). It was administrated orally three consecutive days with 5 mg kg^-1^ dose and until inoculation bacteria 1 mg kg^-1^ dose (a maintenance dose) to rabbits. Given that any manipulation of the cornea is painful, in order to comply with ethical principles and rules of animal rights as well as to prevent animal heave during the process of creating keratitis, anesthesia induction was performed with Ketamine hydrochloride (35 mg kg^-1^ dose, Worden, Holland) and Xylazine hydrochloride (3.5 mg kg^-1^ dose, Worden, Holland). Also, one drop Lidocaine (Aburaihan, Iran) was prescribed in each eye in order to numb the cornea. Using an insulin syringe with a needle size 30, ten microliters of bacterial suspension (10^5^ CFU/mL) were inoculated into each cornea. The needle was placed horizontally and tangent in the corneal surface and injected about 0.2 mm into the corneal stroma. Fifteen to 20 minutes late rabbits regained consciousness. 

*Treatment groups*

The rabbits were randomly divided into eight equal groups. The medication in each group was as follows: 

CO −: Negative control group, rabbits without bacterial inoculation; CO +: Positive control group, treated using with saline; C: treated using with Ciprofloxacin; N: treated using with Ag-NPs; C+B: treated using with Ciprofloxacin and Betamethasone; N+B: treated using with Ag-NPs and Betamethasone; C+N: treated using with Ciprofloxacin and Ag-NPs; C+N+B: treated using with Ciprofloxacin, Ag-NPs and Betamethasone.Twelve hours after bacterial inoculation four times daily treatment of groups were done (according to the institutional ethical committee guidelines). In fact, the duration of treatment was 108 post infection for four rabbits in each group and 204‌h for the rest of the rabbits.

*Clinical evaluation of experimental Pseudomonas keratitis*

Twelve h after bacterial inoculation, daily eye exams and evaluating clinical symptoms were done. Three clinical factors were considered in this study: the number of days of ocular discharge, and blepharospasm, and also corneal opacity. Corneal opacity was scored based on 0-3 with a decreasing trend at 12, 108, and 204 h after bacterial inoculation. The rating system used to grade corneal opacity was in this way: grade 0, Lack of corneal opacity; grade 1, faint opacity; grade 2, Iris fade; grade 3, and Lack of pupil (16). 

*Bacterial count*

Four rabbits of each group were euthanized using CO_2 _at108 and 204‌h after inoculation following general anesthesia with Xylazine and Ketamine. The eyes were bugged out with a gap in the middle of the eye by scissors under sterile conditions. Then the corneas were dissected from the eyes with a sterile scalpel blade and homogenized in one mL of 1% protease peptone. After vortexing to determine the number of bacteria (CFU) per milliliter, 10 µL of the each homogenized cornea were diluted 1:10000 in sterile saline. Then, 100 µL of each diluted sample was plated in triplicate on Mueller Hinton agar medium and incubated for 24 h at 37 °C (6). The plates that had 30 to 300 bacterial colonies were counted and the average results of each cornea were reported as log_10_ CFU mL^-1^.

**Data statistical analysis**

Data analyzing was done by SPSS statistical software, version 18. Scoring corneal opacity results of the groups were analyzed statistically by Kruskal-Wallis test. Also, Mann-Whitney U-test was used to compare differences between two groups. One-way analysis of variance (ANOVA) was performed on the number of days of blepharospasm, ocular discharge, and bacterial counts. Tukey’s test was used to find out the difference between the different groups in the event the (ANOVA) was significant. Also, P value less than 0.05 was significant. 

## Results

*MIC and MBC determination*

The results showed that the MIC and MBC of Ag-NPs in combination with Ciprofloxacin were less than each of them alone (Table 1).

*Clinical evaluation of experimental Pseudomonas keratitis*

Twelve h post inoculation, all eyes were infected. The mean rank of opacity scores of all infected rabbits weren’t significantly different between any of the groups at this time (Kruskal-Wallis, *P* > 0.05) (Figure 1). Also, the differences between the means ± standard deviation of the number of days of blepharospasm and ocular discharge of all treatment groups except CO − weren’t significant in the first 48 h (ANOVA, *P* > 0.05) (Figure 2).

At the end of 108 and 204 h, corneal opacity in the C+N+B group was better than others that it was significantly less than that observed in groups CO +, CO −, N and N+B (Mann-Whitney U-test, *P* < 0.05) (Table 2 and Table 3, Figure 2).

Also, the means number of days of blepharospasm and ocular discharge in C+N+B group were significantly less than other groups at the end of treatment period. Of course, at 204 h post infection it wasn’t significant between groups C+N+B and C+B for ocular discharge (Table 4 and Table 5).

*Bacterial count*

ANOVA showed significant difference among the eight groups at 108 and 204 h (*P* < 0.0005) (Table 6). The mean bacterial count **(**log CFU mL^-1^) in C+N+B group was less than others except CO −. According to post-hoc test it was significantly different with CO +, N and N+B groups at 108 and 204 h. Also, there were no significant difference between CO − and C+N+B at 108 h and CO − with C+N+B and C+N at 204 h post infection (Table 7).

## Discussion

*Pseudomonas aeruginosa* is an opportunistic pathogen which can cause severe corneal infections (17). It can quickly produce progressive necrosis of the corneal stroma (18). Unlike other mucosal surfaces, healthy cornea has no blood and lymph vessels and immune system (19). Any injury to the corneal epithelium makes it susceptible to acute infections (20). Hosting features, such as the immune system status as well as microbial virulence factors are important in the incidence of corneal infections. Fluoroquinolones have excellent *in-vitro* activity against a wide range of Gram-negative and Gram-positive organisms (21). Due to the consequences of long-term antibiotic use, it is a critical need to identify new antibacterial compounds and new method to prevent and treat bacterial infections. Onlen *et al*. used Propolis in the treatment of experimental *Pseudomonas* keratitis in rabbit. They said: “Our results revealed that Propolis has a very limited effect when used alone on both bacterial count and corneal opacity compared with any of drug combinations involving Ciprofloxacin on experimental rabbit model of *P. aeruginosa* keratitis” (6). In our results it was also observed that Ag-NPs cannot be a replacement for antibiotic, although combination of them had a positive strong influence on variables assessed especially in the presence of Betamethasone. *In-vitro* antibacterial effects of Ag-NPs against *Pseudomonas aeruginosa* were confirmed by various studies (22-26). Similar to other studies (27-29) the MIC and MBC results of Ag-NPs in combination with Ciprofloxacin in this study were less than using each of them alone. In some studies, Ag-NPs were combined with other materials in order to enhance their antibacterial activity. For example, combination of Ag-NPs and Chitosan acetate were evaluated against *Pseudomonas aeruginosa *in burn patients. This product showed significant inhibitory effects in preventing infections caused by *Pseudomonas aeruginosa* (30). Also, Mirzaei *et al*. reported that Allicin with Ag-NPs had synergistic effect against skin infection caused by *Pseudomonas aeruginosa *(31). The acquisition of antibiotics resistance by *Pseudomonas aeruginosa* is a concern of exclusive antibiotic treatment. Using Ag-NPs in combination with an antibiotic can be a good idea to prevent bacterial resistance. A large number of studies’ results demonstrated synergistic effect of Ag-NPs in combination with antibiotics against bacteria especially *Pseudomonas aeruginosa* (28, 32, 33). In our results at the end of treatment period bacterial counts (log CFU mL^-1^) in C+N+B and C+N groups were less than others except CO – and also these groups did not have any significant differences with CO – group. It can confirm synergistic effect of combination Ag-NPs and Ciprofloxacin against *Pseudomonas aeruginosa PAO1* in *in-vivo* condition. Using of Ag-NPs to treat eye infections less has been done in the past. One reason may be obscurity about the toxic effects of Ag-NPs on mammalian eye. Santoro *et al*. emphasized that it depends on Ag-NPs diameter size. So that Ag-NPs with a large size (40 nm and larger) did not have significant toxicity on the eye cells and other mammalian cell lines (34). 

## Conclusion

The results of the present study do not allow any final conclusions. To this aim, more studies of Ag-NPs ocular side effects as an adjutant in keratitis treatment must be done. However, our results confirmed that the combination of Ag-NPs with a specific antibiotic such as Ciprofloxacin have a positive synergistic influence on *Pseudomonas *keratitis treatment. According to corneal opacity scores, the number of days of blepharospasm, and ocular discharge, it seems that keratitis treatment process in C+N+B group was better than traditional treatment method with Ciprofloxacin and Betamethasone. It seems that using combination of them against *Pseudomonas aeruginosa* has the following advantages: 1. Enhancement of the their antibacterial effects and as a result decrease inthe therapeutic dose of each of them in compared to using either alone. 2. Decrease in the genetic mutations probability in bacteria against antibiotic in combination with Ag-NPs and thus decrease in the emergence of resistant *Pseudomonas* strains. 
